# A training manual for event history data management using Health and Demographic Surveillance System data

**DOI:** 10.1186/s13104-017-2541-9

**Published:** 2017-06-26

**Authors:** Philippe Bocquier, Carren Ginsburg, Kobus Herbst, Osman Sankoh, Mark A. Collinson

**Affiliations:** 1 Centre de Recherche En Démographie, Université Catholique de Louvain, Louvain‐la‐Neuve, Belgium; 2 Medical Research Council/Wits Rural Public Health and Health Transitions Research Unit (Agincourt), School of Public Health, Faculty of Health Sciences, University of the Witwatersrand, 27 St Andrews Road, Parktown, Johannesburg, 2193 South Africa; 3 Africa Health Research Institute, University of KwaZulu-Natal, Durban, South Africa; 4 INDEPTH Network, Accra, Ghana; 5 School of Public Health, Faculty of Health Sciences, University of the Witwatersrand, Johannesburg, South Africa; 6 Department of Mathematics and Statistics, Njala University, Njala, Sierra Leone; 7 Umeå Centre for Global Health Research, Umeå University, Umeå, Sweden

**Keywords:** Longitudinal data management, Event history analysis, Health and Demographic Surveillance System

## Abstract

**Objective:**

The objective of this research note is to introduce a training manual for event history data management. The manual provides a first comprehensive guide to longitudinal Health and Demographic Surveillance System (HDSS) data management that allows for a step-by-step description of the process of structuring and preparing a dataset for the calculation of demographic rates and event history analysis. The research note provides some background information on the INDEPTH Network, and the iShare data repository and describes the need for a manual to guide users as to how to correctly handle HDSS datasets.

**Results:**

The approach outlined in the manual is flexible and can be applied to other longitudinal data sources. It facilitates the development of standardised longitudinal data management and harmonization of datasets to produce a comparative set of results.

**Electronic supplementary material:**

The online version of this article (doi:10.1186/s13104-017-2541-9) contains supplementary material, which is available to authorized users.

## Introduction

The International Network for the Demographic Evaluation of Populations and their Health (INDEPTH) was founded in 1998 and represents a group of currently 47 Health and Demographic Surveillance System (HDSS) sites located in 18 low- and middle-income countries in Africa, Asia and the Pacific. Following its establishment, the Network has endeavoured to build a standardised set of data management protocols pertaining to HDSS data [1]. One of the key challenges of HDSS data management relates to the effective and efficient means of storing and maintaining longitudinal data on health, socio-economic and demographic dynamics that are prospectively updated within a geographically-defined population. These longitudinal datasets capture the dynamic sets of events and episodes pertaining to every individual and household under surveillance (including migration into and out of the demarcated HDSS area). They require sound database structures and protocols for data management and storage. The HDSS platforms form the backbone for high-quality data analysis for scientific enquiry and embedding research projects.

One of the research priorities of the INDEPTH Network is to facilitate comparative demographic analyses across HDSSs. The Multi-centre Analysis of the Dynamics of Internal Migration And Health (MADIMAH) is an INDEPTH project that commenced in 2011 with the aim of producing a set of comparative analyses across HDSSs on questions concerning migration and health [2]. The first phase of MADIMAH involved a study of migration, urbanisation and human capital using datasets from eight HDSSs in Burkina Faso, Kenya, South Africa and Mozambique [3]. Thereafter, a multi-centre study examining the migration effect on mortality across nine sub-Saharan African HDSSs was conducted [4]. These studies illustrate the use of standardised longitudinal data management and harmonization of HDSS data across multiple centres. They further illustrate the scientific potential that may be realised by following a uniform analytical framework to produce a comparative set of results across different locations. The objective of this research note is to introduce a training manual for event history data management which proposes a set of versatile procedures aimed at producing standard data structures for use in longitudinal event history analysis (EHA).

## Main text

### Data

The INDEPTH iSHARE2 data repository^1^ went online in 2014 and provides a unique resource of high-quality, fully documented HDSS longitudinal datasets available for download to a wide range of users, including HDSS-linked scientists and analysts, researchers and students [5]. The repository, which is growing over time, holds amongst others, core micro datasets describing the key demographic events of more than 25 HDSS populations and unique data on cause specific mortality [5]. Recently, the first of a series of multi-centre core micro datasets attached to the MADIMAH project has been released and is structured to examine determinants of in- and out-migration, particularly the education status of the migrant [6].

### Methods

The efficient use of these micro datasets requires that users are able to handle HDSS data structures (such as the residency episode files) and understand the range of core events that alter residency status in the HDSS, especially, in- and out-migration, births and deaths. These data structures and properties form the necessary foundation for the statistical analyses of population dynamics. In order to address these requirements, the MADIMAH group developed a manual based on the group’s experiences of conducting comparative analyses across multiple HDSS sites, and of training HDSS data scientists and analysts in these methods. The intention was to provide data managers and analysts who manage raw questionnaire data with a step-by-step description of the process of structuring and preparing a dataset for the calculation of demographic rates and EHA. The approach was to create a common language and set of codes that can create synergies and enable communication across larger communities of data managers and analysts working with longitudinal research designs.

### Results: training manual

The training manual is available on-line as Additional file 1 to this note. It provides a general introduction to event history data management. The manual leads the user through all the procedures necessary to format and analyse longitudinal data. It demonstrates how to create a core residency file suitable for EHA and how to check for inconsistencies in the data. The approach is flexible and covers the calculation of basic demographic rates, as well as more complex determinants analysis through the addition of individual and household attributes. The manual illustrates how to enrich the database with new events with precise or imputed dates of occurrence. Finally, the manual explains how to create duration events of several types. The methods outlined in the manual are implemented in detailed coding using Stata software. All sections start with an example of an output file, followed by a check-list and conclude with further examples or programmes needed to solve specific technical issues. Longer, more detailed Stata programmes are available in Additional file 1: Appendix.

This manual is the first comprehensive guide to HDSS longitudinal data management and has become a standard for INDEPTH member HDSS Centres. It can be implemented on longitudinal data from other sources, including register-based, retrospective, or cohort data. It forms the first part of a two-part series. The second manual will guide analysts through the computation of demographic rates and the analysis of determinants and outcomes of demographic processes, using the longitudinal dimension in the data.

## Limitations

The procedures outlined in the training manual are most comprehensively applied to HDSS data because these data are inclusive of all entry and exit events in a geographically defined population. In other data sources some entry or exit events might not be relevant, e.g. in-migration for cohort data, or death for retrospective data. Nonetheless, the procedures described in this manual will remain valid and only minor changes to the programming codes will be necessary to apply these methods to such study designs.

## Additional file

**Additional file 1.** Manual of event history data management using HDSS data.

## Abbreviations

INDEPTH: International Network for the Demographic Evaluation of Populations and their Health
HDSS: Health and Demographic Surveillance System
EHA: event history analysis
MADIMAH: Multi-centre Analysis of the Dynamics of Internal Migration And Health

## Footnotes

1
http://www.indepth-ishare.org/index.php/home.
