# Supplementary material for: A training manual for event history data management using Health and Demographic Surveillance System data
Source: BMC Res Notes. 2017 Jun 26;10:224. doi: 10.1186/s13104-017-2541-9 (PMC5485641; doi:10.1186/s13104-017-2541-9)
Supplement: Supplementary file 1 — Additional file 1. Manual of event history data management using HDSS data. [file 13104_2017_2541_MOESM1_ESM.pdf]

Manual of
Event History Data Management using HDSS data

Philippe Bocquier and Carren Ginsburg

Foreword

The Migration, Urbanisation and Health Working Group (MUHWG), hosted by the International Network for the Demographic Evaluation of Populations and Their Health (INDEPTH) has advised on a longitudinal data format based on an event history analysis (EHA) approach used in the Multi-centre Analysis of the Dynamics of Internal Migration and Health (MADIMAH) project. This format is the basis of the micro-data specification that has now become a standard for all INDEPTH member Health and Demographic Surveillance Systems (HDSS). This is a flexible format that lends itself to the calculation of basic demographic rates, but it is also suitable for more thorough EHA. The basic standard – also named core residency file – can be easily expanded through the addition of event attributes or other status events, e.g. educational attainment, employment status, anthropometry measurements, etc.

This manual presents the different longitudinal data management steps, commencing with the creation of the core residency file through to producing the most sophisticated event history file. The proposed procedures are aimed at producing a standard data structure that can be understood and implemented whatever the nature of the longitudinal data, whether HDSS, register, retrospective, or cohort data. However, because they cover all entry and exit events in a geographically defined population, HDSS data are the most comprehensive and will be taken as a gold standard. In other data sources some entry or exit events might not be relevant, e.g. in-migration for cohort data, or death for retrospective data. Nonetheless, the rationale described in this manual will remain valid and only minor changes to the programming codes will be necessary in such instances.

Following a glossary and a general introduction to event history data management, the manual is structured as follows:

- The first section demonstrates how to create a core residency file suitable for event history analysis.
- The second section illustrates how to add fertility events (deliveries) as an example of an event that does not modify the residency status of the individuals.
- The third section indicates how to add other events that may entail the imputation of dates.
- The last and fourth section explains how to create duration events of several types.

All sections start with an example of an output file, followed by a check-list, and conclude with further examples or programmes needed to solve some technical issues. Longer programmes are available in Annexes.

This manual aims to guide the derivation of a longitudinal data file that is suitable for event history analysis. The analytical techniques themselves are the subject of a second manual that focuses on the

analysis and production of indicators of renewable events (migrations, fertility) and non-renewable events (mortality).

Acknowledgement

We would like to acknowledge scientific contributions of Dr. Mark A. Collinson, Dr. Donatien Béguy, Dr. Kobus (A.J.) Herbst, Dr. Sulaimon Afolabi, and Baptiste Beck. The INDEPTH Multi-centre Analysis of the Dynamics of Internal Migration And Health (MADIMAH) project has received funds from the Swedish International Development Agency (Sida: 2012-000379) The research has received a joint financial support from the National Research Foundation, South Africa, and the Wallonia-Brussels Federation of Belgium (Grant No: 95284).. We further acknowledge institutional support from the School of Public Health, Faculty of Health Sciences, University of the Witwatersrand, South Africa; Centre de Recherche en Démographie et Sociétés, Université Catholique de Louvain, Louvain-la-Neuve, Belgium; and the African Population and Health Research Centre, Nairobi, Kenya, as critical bases for the MADIMAH project leadership. We gratefully acknowledge the South African Medical Research Council (SAMRC) for funding Carren Ginsburg's Career Development Award.

Table of contents

1 Introduction 6

2 Constructing the core residency file 6

2.1 Example of residency episode file as an input file 9

2.2 Example of core residency file 10

2.3 Check-list for core residency file 12

2.4 Time in millisecond 12

2.5 Creating OBE using “stsplit” command after “stset” 13

2.6 Producing and interpreting the event consistency matrix 13

2.7 Stata programmes 15

2.7.1 Step 1: Converting episode file into core residency file 15

2.7.2 Step 2: Creating a common censoring date for all individuals 16

2.7.3 Step 3: Creating the consistency matrix 16

2.7.4 Step 4: Creating a residence variable 16

3 Adding events linked to ego, e.g. fertility events 18

3.1 Example of EHA file with birth history 19

3.2 Procedure to add fertility events 20

3.2.1 Step 1: Duplicate file A: 20

3.2.2 Step 2: Create file B: 20

3.2.3 Step 3: Merge file A and B into file C according to time using “tmerge.ado” (see Annexes): 21

4 Adding other biographical events defining changes of status 22

4.1 Example of EHA file with change of status 22

4.2 Procedure to add change of status 23

4.2.1 Step 1: Create file for change of status 23

4.2.2 Step 2: Merge file A and B into file C according to time using “tmerge.ado” (see Annexes): 24

5 Adding duration events 25

5.1 Duration specific to individual’s own biography 25

5.1.1 Create in-migration status 26

5.1.2 Create duration of residence out of the HDSS 27

5.1.3 Create a variable identifying new in-migrant and return migrant 28

5.1.4 Create a variable combining the different status 28

5.2 Calendar periods 29

5.2.1 Split time into calendar period 29

5.2.2 IMPORTANT: Censoring variables have to be recomputed after each stplit 30

5.3 Age groups 30

5.3.1 Split time into age group 31

5.3.2 IMPORTANT: Censoring variables have to be recomputed after each stplit 31
6 Appendix 32
6.1 tmerge program 32
6.2 tmerge help menu 33

Event history glossary as used in this manual (in alphabetical order)

Censoring (right-): time from which the individual is no longer observed

Characteristic: variable denoting an attribute of an individual that does not change over time (e.g. sex, birth year)

Core event: events that change the residency status of the individual (such as: enumeration, birth, death, in-migration, out-migration, end of observation)

Core residency file: a standardised file format containing the core events for each individual in the study/surveillance population, each event being recorded on a single record

Cross-sectional data: data on characteristics and statuses with no reference to time change and usually collected at a single point in time

Event consistency matrix: a matrix that crosses preceding events with the current events in order to check for any inconsistencies in the core residency file

Event history analysis (EHA): statistical regression analysis technique that identifies the effects of fixed and time-varying covariates on the time of occurrence of an event in presence of censoring

Incidental event: events that do not change the individual's residency status (e.g. change of household within the surveillance area; change of educational level…)

Long format file: a file where events and corresponding time points are recorded on different lines and linked with an individual identifier

Longitudinal data (or event history data): data on characteristics, changes of status, and events with corresponding dates pertaining to each individual

Panel data: a set of cross-sectional data collected from the same individuals at different points in time

Prospective data: regular rounds of data collection on events or changes of status that occurred since the last round of data collection

Register: an official list of individuals in a population updated at each core event

Residency episode file: a file where each episode of residence is marked with a start event and an end event in a single record with their corresponding time points

Retrospective data: data derived from questions concerning past events or changes of status starting from birth or from another fixed point in time

Status: variable denoting an attribute of an individual that may change over time (e.g. education level, BMI)

Surveillance system: a population registration system that monitors health and demographic dynamics in a geographically defined population through prospective data collection

Survey data: information gathered from a set of questions posed to a sample of respondents

Survival analysis (also named time-to-event analysis, failure time analysis, duration analysis): descriptive statistical analysis technique that examines the time of occurrence of an event in presence of censoring

Truncation: time until which the occurrence of an event is not known

Wide format: events and corresponding time points are recorded on one line per individual

1 Introduction

Computing individual exposure and basic rates requires a standard format core residency file. In the case of the INDEPTH Network, this format allows member Centres to provide surveillance data in a standard way for repository and analytical purposes. However, the proposed longitudinal data format is not limited to the use of HDSS data: it is applicable to any type of longitudinal data, i.e. data obtained from surveillance, registers or from retrospective or panel surveys. In fact, much data collection conducted by INDEPTH Network member Centres involve part surveillance data (often in the form of retrospective data), part survey data (often in the form of panel data).[1]

In addition to its first goal, computing rates and conducting analysis, event history analysis or core residency file format allows for the calculation of longitudinal data quality metrics:

1. The very process of creating the core residency file first involves checking basic inconsistencies on dates and types of events: out-of-range values, coding errors, unusual frequencies, etc.
2. The second and most crucial step in checking quality involves the construction of a matrix crossing (current) events with following-events, referred to as the event consistency matrix to check the coherence of event sequences. Ordering of events is easily checked through this matrix and details of order inconsistencies can be produced to make corrections in the base files before event history data is processed further.
3. The first and second steps are internal consistency checks. The third step involves computing the basic rates defining the entry and exit from the population under study and checking these rates against known trends (external consistency checks) from the same population, from similar populations or from larger spatial units (region, country). These basic rates are birth and in-migration rates, and death and out-migration rates, in the context of surveillance systems or registers. It is usually not possible to check these rates for retrospective data sources because the dead and the emigrants are not accounted for.

Employing a standardised format facilitates pooling data for multi-site analysis. It further allows for variable naming, labelling of categorical variables and display formatting to be applied consistently. Standardisation helps create a common language and enables the sharing of codes for data management and analysis. The longitudinal data structure being the same, statistical programmes can be understood by a large community.

2 Constructing the core residency file

The surveillance data associated with a particular individual over the course of her exposure to demographic surveillance are represented by a series of event records. The first event for any individual will be either enumeration, birth, or in-migration. This will be followed by a number of records corresponding to each observed event involving that individual, such as out-migration, death, or end of observation (censoring). All these basic events define entry or exit from a population under observation, i.e. a population defined within time and spatial boundaries. In other words, the basic events define the residency status of each individual recorded in the database: this is why the longitudinal data comprising these events is named the “core residency file”. Each event has a set of standard attributes (Table 1) that are common to all events, followed by a series of attributes specific to the event. All variables in bold font are compulsory for inclusion in the core residency file.

---

[1] A set of specific terms used in this manual are defined in a glossary presented at the beginning of this document.

TABLE 1 : COMMON EVENT ATTRIBUTES

Attribute	Variable Name	Description	Comment
Record Number	*RecNr*	*A sequential number uniquely identifying each record in the data file*	*Optional: mainly for repository purposes*
Country Identifier	CountryId	ISO 3166-1 numeric code of the country in which the surveillance site is situated	Mainly for multi-site analysis purposes
Centre Identifier	CentreId	An identifier issued by INDEPTH to each member centre of the format CCCSS, where CCC is a sequential centre identifier and SS is a sequential identifier of the site within the centre in the case of multiple site centres	Mainly for multi-site analysis purposes
Location Identifier	LocationId	Unique identifier associated with a residential unit within the site and represents the location where the individual was or became resident when the event occurred	For data anonymisation purposes, this identifier should not be the same as the identifier used internally by the Centre. However, the Centre should be able to map this identifier to their original identifier
**Individual Identifier**	**IndividualId**	**A number uniquely identifying all the records belonging to a specific individual in the data file**	**For data anonymisation purposes, this identifier should not be the same as the identifier used internally by the Centre. However, the Centre should be able to map this identifier to their original identifier**
**Date of birth**	**DoB**	**The date of birth of the individual**	**Preferably in Stata `%tc` format in double precision (time in milliseconds)**
**Sex**	**Sex**	**Sex of the respondent**	**1:Male; 2:Female**
**Event**	**EventCode**	**A code identifying the type of event that has occurred**	**Cf. Table 2**
**Event date**	**EventDate**	**The date on which the event occurred**	**Preferably in Stata `%tc` format**
Observation date	ObservationDate	Date on which the event was observed (recorded), also known as surveillance visit date	This variable is useful in the creation of EventCode = 18 (see Table 2)
Event count	*EventCount*	*The total number of events associated with this individual in this dataset*	*Optional: mainly for repository purposes*
Event number	*EventNr*	*A number increasing from 1 to EventCount for each event record in order of event occurrence*	*Optional: mainly for repository purposes*
**Residence**	**residence**	**A binary variable indicating exposure or residency status in the population (see Stata program 3)**	**0:no; 1: yes**

NB: **All variables in bold font are compulsory**. Variables in normal font are advisable. *Variables in italic font are optional.*

Table 2 defines the possible events referred to in Table 1 as EventCodes. These fall into two categories, namely:

- Core events that change the residency status of the individual and are necessary to construct the core residency file. These codes appear in the Table in bold font and are compulsory.
- Incidental events that do not change the residency status and add observations to the core residency file.

TABLE 2: EVENT CODES AND DEFINITION (CF. VARIABLE EVENTCODE IN TABLE 1)

Event	Code	Definition	Attributes	Attribute Description
**Enumeration**	**ENU = 1**	**Starting event for all individuals present at the baseline census of the surveillance area. It corresponds to the date on which the individual was first observed to be present in the surveillance area during the baseline census.**		
**Birth**	**BTH = 2**	**The birth of an individual to a resident female.**	**MotherId**<br>*DeliveryId*	**The IndividualId of the mother.**<br>*When available, equals the LBCnt (live birth count) associated with this birth.*
**In-migration**	**IMG = 3**	**The event of migrating into the surveillance area.**	**Origin**	**Classification scheme according to study's need (e.g. capital city, next big city, other cities, towns, other rural, foreign urban, foreign rural)**
**Out-migration**	**OMG = 4**	**The event of migrating out of the surveillance area.**	**Destination**	**(same as for Origin)**
Location exit	EXT = 5	The event of leaving a residential location within the surveillance area to take up residence in another residential location within the surveillance area.	LocationId_dest	The LocationId of the destination location within the surveillance area to which the individual moved.
Location entry	ENT = 6	The event of taking up residence in a residential location within the surveillance area following a location exit event. Note that location exit and entry are actually two parts of the same action of changing residential location, and as such they happen on the same event date.	LocationId_orig	The LocationId of the residential origin location within the surveillance area from which the individual moved.
**Death**	**DTH = 7**	**The death of the individual under surveillance. The date of death is the event date.**	**Cause1**<br>**Cause2**<br>**Cause3**<br>**Likelihood1**<br>**Likelihood2**<br>**Likelihood3**	**Up to three causes of death coded using the WHO list of verbal autopsy death causes. Likelihood values associated with each possible cause of death*.**
**Observation end**	**OBE = 9**	**An event inserted when a dataset is right censored at an arbitrary date (the individual remains under surveillance beyond this date). The right censor date is the date of this event.**		
Delivery	*DLV = 10*	*The event of a pregnancy ending after 28 weeks of gestation, which may or may not result in the birth of one or more individuals (represented in this dataset by a BTH event linked to this delivery event).*	*LBCnt*<br>*SBCnt*<br>*Parity*	*Live birth count*<br>*Stillbirth count*<br>*The number of live births to this woman prior to the current delivery.*
Observation	OBS = 18	Used to record characteristics of individuals under surveillance valid at the time of the observation, e.g. educational attainment, employment status or anthropometry measures. These events are not part of the minimum core individual dataset, but their inclusion may be necessary for analysis. Note that their values may change over time.		

Last Observation	*OBL = 19*	*An event indicating the last point in time that this individual was observed to be present in the surveillance population. Event date equals observation date in this instance. Normally there should be no individuals with this event as their last event if the right censoring date is prior to the start of the last complete census round.*		
Imputation	*IPT = 20*	*Imputed date of change in status recorded at the time of observation when the exact date of change is not known. Other codes (21, 22…) could be attributed to a specific type of change (e.g. 21=matrimonial status; 22=employment status; etc.).*		
Period	*PER = 30*	*An event that marks the change from one period to the next as defined by the user. Other codes (31, 32…) could be attributed to a specific type of period (e.g. 31=decade; 32=season; etc.).*		
Age group	*AGE = 40*	*An event that marks the change from one age group to the next as defined by the user. Other codes (41, 42…) could be attributed to qgrouping (e.g. 41=0,1,5,10…70; 42=15,25,35…85; etc.).*		

NB: **All codes in bold font are compulsory**. Codes in normal font are advisable. *Codes in italic font are optional.*
*: cf. International Classification of Diseases (ICD) http://www.who.int/classifications/icd/en/

2.1 Example of residency episode file as an input file

Table 3 presents an example of an episode file, with start event and end event in each record. The entries in italic font, i.e. after an individual's death or out-migration, do not usually appear in the original record. When no event occurred by the end of observation, the variables EndEventCode and EndEventDate might be missing (individual A). It is necessary to define a censoring date, i.e. a date at which observation ends (here 31 Dec 2010) and to create an extra record spanning the time between the last recorded event and the end of observation date. In the Stata programme 2 (2.5.2), the same censoring date is proposed for all individuals, but this consistency across individuals is not absolutely necessary (although much easier to manage). What is absolutely necessary is that for a particular individual, the censoring date is the same in all data files.

Table 3: Example of a Residency Episode File

	**IndividualId**	LocationId	**DoB**	**StartEvent Code**	**StartEvent Date**	**EndEvent Code**	**EndEvent Date**
A	A0010010010001	A00100100100	17 Nov 1947	ENU	21 Aug 2002	*OBE*	*31 Dec 2010*
B	A0010010010002	A00100100100	1 Jul 1976	ENU	21 Aug 2002	OMG	1 Jul 2007
	A0010010010002	*A00100100100*	*1 Jul 1976*	*OMG*	*1 Jul 2007*	*OBE*	*31 Dec 2010*
C	A0010010010003	A00100100100	23 Aug 1985	ENU	21 Aug 2002	EXT	1 Jul 2007
	A0010010010003	A00203000104	23 Aug 1985	ENT	1 Jul 2007	OMG	10 Nov 2007
	A0010010010003	A00100100111	23 Aug 1985	IMG	30 Mar 2009	DTH	15 Oct 2009
	A0010010010003	*A00100100111*	*23 Aug 1985*	*DTH*	*15 Oct 2008*	*OBE*	*31 Dec 2010*
D	A0010010010004	A00100100100	1 Jul 1988	ENU	21 Aug 2002	EXT	1 Jul 2007
	A0010010010004	A00203000104	1 Jul 1988	ENT	1 Jul 2007	OMG	10 May 2008
	A0010010010004	*A00203000104*	*1 Jul 1988*	*OMG*	*10 May 2008*	*OBE*	*31 Dec 2010*
E	A0010010010005	A00100100100	1 Jul 2005	BTH	1 Jul 2005	OBE	31 Dec 2010
F	A0010010010006	A00100100100	1 Jul 1983	IMG	31 Aug 2007	OMG	8 Apr 2010
	A0010010010006	*A00100100100*	*1 Jul 1983*	*OMG*	*8 Apr 2010*	*OBE*	*31 Dec 2010*

Comments on each of the 6 individuals A to F:

A. Enumerated at the start of the HDSS, still in the HDSS on 31 Dec 2010.
B. Enumerated at the start of the HDSS, out-migrated from the HDSS on 1 Jul 2007. Note that the last observation is the end of observation, i.e. the same right-censoring time for all individuals, 31 December 2010.
C. Enumerated at the start of the HDSS, changed household within the HDSS on 1 Jul 2007 (a one-day lag was artificially created between 'exit' and 'entry' into the new household - you may choose to add

just a few hours instead of a day). The individual then out-migrated from the HDSS on 10 Nov 2007, in-migrated into the HDSS on 30 Mar 2009 (i.e. more than 6 months after out-migrating), and died in the HDSS 15 Oct 2009. End of observation is again 31 Dec 2010.

D. Enumerated at the start of the HDSS, changed household within the HDSS on 1 Jul 2007 (a one-day lag was artificially created between 'exit' and 'entry' into the new household), out-migrated from the HDSS on 10 May 2008 (you may choose to add just a few hours instead of a day). End of observation is 31 Dec 2010.

E. Born in the HDSS 1 Jul 2005, still in the HDSS on 31 Dec 2010.

F. In-migrated into the HDSS on 31 Aug 2007, out-migrated from the HDSS on 8 Apr 2010 (i.e. more than 6 months after in-migrating). End of observation is 31 Dec 2010.

2.2 Example of core residency file

Table 4 presents an example of a basic EHA file, i.e. a core residency file. Extracted from the original "residency episode file" (with start and end events for each exposure episode), the following core residency file is obtained by sorting recorded events for each individual by dates of occurrence (Stata programme 1). Note that all records for an individual end with an OBE (end of observation) record. After defining a censoring time (OBE) for all individuals (Stata programme 2), the "residence" variable can be computed (Stata programme 3). This file format is called the long format.

TABLE 4: EXAMPLE OF A CORE RESIDENCY FILE

	**IndividualId**	LocationId	**DoB**	**EventCode**	**EventDate**	**residence**
A	a0010010010001	A00100100100	17 Nov 1947	ENU	21 Aug 2002	0
	a0010010010001	A00100100100	17 Nov 1947	OBE	31 Dec 2010	1
B	A0010010010002	A00100100100	1 Jul 1976	ENU	21 Aug 2002	0
	A0010010010002	A00100100100	1 Jul 1976	OMG	1 Jul 2007	1
	A0010010010002	A00100100100	1 Jul 1976	OBE	31 Dec 2010	0
C	A0010010010003	A00100100100	23 Aug 1985	ENU	21 Aug 2002	0
	A0010010010003	A00100100100	23 Aug 1985	EXT	1 Jul 2007	1
	A0010010010003	A00203000104	23 Aug 1985	ENT	1 Jul 2007	1
	A0010010010003	A00203000104	23 Aug 1985	OMG	10 Nov 2007	1
	A0010010010003	A00100100111	23 Aug 1985	IMG	30 Mar 2009	0
	A0010010010003	A00100100111	23 Aug 1985	DTH	15 Oct 2009	1
	A0010010010003	A00100100111	23 Aug 1985	OBE	31 Dec 2010	0
D	A0010010010004	A00100100100	1 Jul 1988	ENU	21 Aug 2002	0
	A0010010010004	A00100100100	1 Jul 1988	EXT	1 Jul 2007	1
	A0010010010004	A00203000104	1 Jul 1988	ENT	1 Jul 2007	1
	A0010010010004	A00203000104	1 Jul 1988	OMG	10 May 2008	1
	A0010010010004	A00203000104	1 Jul 1988	OBE	31 Dec 2010	0
E	A0010010010005	A00100100100	1 Jul 2005	BTH	1 Jul 2005	0
	A0010010010005	A00100100100	1 Jul 2005	OBE	31 Dec 2010	1
F	A0010010010006	A00100100100	1 Jul 1983	IMG	31 Aug 2007	0
	A0010010010006	A00100100100	1 Jul 1983	OMG	8 Apr 2010	1
	A0010010010006	A00100100100	1 Jul 1983	OBE	31 Dec 2010	0

Below are comments on each of the 6 individuals A to F. A graph attached to each example shows the lifeline of each individual between enumeration and right-censoring time. A solid line means the individual is in the HDSS, a dashed line means the individual is out of the HDSS:

A. Enumerated at the start of the HDSS, still in the HDSS on 31 Dec 2010.

ENU OBE

A |____|

21/08/2002 31/12/2010

B. Enumerated at the start of the HDSS, out-migrated from the HDSS on 1 Jul 2007. Note that the last observation is the end of observation, i.e. the same right-censoring time for all individuals, 31 December 2010 in this example.

ENU OMG OBE
B|______|........|

21/08/2002 01/07/2007 31/12/2010

C. Enumerated at the start of the HDSS, changed household within the HDSS on 1 Jul 2007 (an 12-hour lag was artificially created between 'exit' and 'entry' in the new household; see section 2.4 for details). The individual then out-migrated from the HDSS on 10 Nov 2007, in-migrated into the HDSS on 30 Mar 2009 (i.e. more than 6 months after out-migrating), and died in the HDSS 15 Oct 2009. End of observation is once again 31 Dec 2010.

EXT/
ENU ENT OMG IMG DTH OBE
C|______|___|........|___|........|

21/08/2002 01/07/2007 10/11/2007 30/03/2009 15/10/2009 31/12/2010

D. Enumerated at the start of the HDSS, changed household within the HDSS on 1 Jul 2007 (a one-day lag was artificially created between 'exit' and 'entry' in the new household), out-migrated from the HDSS on 10 May 2008. End of observation is 31 Dec 2010.

EXT/
ENU ENT OMG OBE
D|______|______|........|

21/08/2002 01/07/2007 10/05/2008 31/12/2010

E. Born in the HDSS 1 Jul 2005, still in the HDSS on 31 Dec 2010.

BTH OBE
E|........|______|

21/08/2002 01/07/2005 31/12/2010

F. In-migrated in the HDSS on 31 Aug 2007, out-migrated from the HDSS on 8 Apr 2010 (i.e. more than 6 months after in-migrating). End of observation is 31 Dec 2010.

IMG OMG OBE
F|........|______|.......|

21/08/2002 31/08/2007 10/05/2008 31/12/2010

2.3 Check-list for core residency file

1. The aim is to get a file with variables as specified in Table 1 (variables in bold font mandatory). The Stata programme 1 (2.5.1) helps to convert an "episode file" (where each episode of residence is marked with a start event and an end event in a single record) into a core residency file where all episodes are ordered according to the date of events.
2. Missing values: make a distinction between 'not applicable' (NA) because of logical conditions (e.g. no education level for children under 6), 'don't know' (DK) as a response category (i.e. the respondent answered but does not know), 'refusal' (RE) as a response category (i.e. the respondent does not wish to answer), and 'missing' which means a response should be recorded but is not due to data collection, data entry, or data processing hazards.
3. The variable EventCode in the EHA file should include:
   a. at least the following events: BTH, ENU, IMG, OMG, DTH, OBE (definitions in Table 2)
   b. preferably internal moves: EXT, ENT (see comment 5 below)
4. All 'hanging cases' (when the residency status or even the survival of a resident absent at the moment of interview is unknown) should have been dealt with. This is why an OBE is compulsory to define the last reliable date of observation (right-censoring date). The Stata programme 2 (2.5.2) is provided to create the censoring observation (OBE) if not readily available in the original dataset. The section below explains how to create an OBE using the "stset" and "stsplit" commands.
5. Some sites do not record internal moves. If they do, an entry ENT should follow an exit EXT, as these codes mark the change of household within the HDSS. An EXT not followed by an ENT is considered a 'hanging case'.
6. It is best to include "events" coded OBS and DLV. However, these events are not required for analysing mortality or migration.
7. To achieve this 'long format', all individuals must have at least two records sorted by date, from the earliest to the latest.
8. After sorting the dataset at the end of Step 2, a consistency matrix described in the next section (2.4) will help identifying inconsistencies in the order of events.
9. The "residence" variable depends on the logical sequence of core events and on the time criterion for residence (Step 4, 2.5.4). The variable "residence" is a binary variable that defines the period of exposure in the population under study, which is essential to all event history analysis. It is constructed using two sources of information:
   - The logical sequence of core events: ENU, BTH, DTH, IMG, OMG, OBE; as well as the following incidental events: EXT, ENT, OBS, OBL (accounted for in the Stata programme).
   - The time criterion for residence, which is specific to each site. Continuous registration systems (such as HDSS, registers etc.) use different time criteria for residency: some use a 1-month duration of residence in the site to be considered a true resident, some use a 6-month duration of residence, some use another time threshold to determine residency. For comparative analysis purposes, it is highly advisable to use a common time criterion. 6 months is generally the preferred time criteria as it has become almost an international standard in demographic analysis.
   - This time criterion applies to entry or exit from the HDSS that is uses to determine residency status. It does not apply to the duration from birth to out-migration or death.

2.4 Time ordering in milliseconds

By default, Stata assumes that successive events should be ordered in time and not simultaneous (a time gap should separate each event from the next). With time in milliseconds (%tc display format, double storage format), all events that were originally entered in days (as usual in HDSS data collection) will be set at 00:00 of that day (i.e. the first second of that day). Stata does not offer a time format in minutes or hours, so it is necessary to set time in millisecond to order events that occurred the same recorded day. It

will be necessary to set a different time of the day for different type of events to avoid confusion for successive events that occurred the same day. The advised procedure is the following:

1. Set all events to 12:00 (i.e. midday)
2. Set date of birth to 12:00
3. Set all event that means entering the population to 06:00 (i.e. ENU, BTH, IMG)
4. If applicable, set EXT event to 06:00
5. Set all event that means exiting the population to 18:00 (i.e. OMG, DTH)
6. If applicable, set ENT event to 18:00

That way, baby who died on the same day they were born will always die 12 hours after they were born. Also, individuals who out-migrated on the same day they in-migrated will always out-migrate 12 hours after they in-migrated. Other events that do not relate to entering or exiting the population (i.e. OBE, DLV, OBS, OBL, IPT, PER, AGE) will be dated at 12:00.

2.5 Creating OBE using "stsplit" command after "stset" command

The easiest way to create an OBE event (end of observation) for all individuals is to use the "stset" command and then the "stsplit" command. The "stset" command is meant for event history analysis: it is creating the censoring variable using the event variable (EventCode) and time variables (EventDate, datebeg) conditional on residence in the HDSS (residence==1).

The program than run "stset" and "stsplit" is in Section 2.6.1 below. Despite time changes explained in the previous section 2.4, it may be that "stset" produces a warning:

```
entry on or after exit (datebeg>EventDate)            PROBABLE ERROR
```

Stata actually means `(datebeg>=EventDate)`. Because `datebeg` should never be greater than `EventDate`, this is an indication that `datebeg` is actually equal to `EventDate` on a particular record, i.e. that two successive records end the same date, at the same hour. The direct consequence is that the record where `datebeg=EventDate` will be discarded. Usually it has no consequence on the analysis (for example when a DLV just occurred the same day as OBS or AGE) but it is important that these potential errors are checked and corrected at least for the event that relate to entering or exiting the population (i.e. ENU, BTH, IMG, OMG, DTH) and for internal moves (i.e. EXT, ENT).

Once the "stset" command has been executed, then the "stsplit" command can be used to set an OBE date for all individuals (see programme in section 2.6.1 for details). After each "stsplit", it is always safe to sort the data again by `IndividualId` and `EventDate` and, at the time of analysis, compute again the censoring variable (see section 5.3.2.).

2.6 Producing and interpreting the event consistency matrix

Beyond errors in dates explained in the previous section, logical errors in the order of events have to be corrected. It is highly probable that when first compiling the dataset some inconsistencies are present in relation to the order of events. The event consistency matrix helps to identify these:

a. The first event should always be ENU, IMG or BTH.
b. The last event should be 'missing' or OBE, OMG or DTH.
c. Common errors include events that occurs twice in a row, e.g. two successive OMG, (a resident would need to IMG before OMG again).
d. Some sequences are impossible, e.g. DTH before BTH, IMG, or OMG.

The Step 3 provides the code for producing the event consistency matrix.

To produce this matrix one needs a variable for the preceding event "EventPrec" that crosses with the current event "EventCode". The program is quite straightforward, however, the interpretation less straightforward. The following matrix will help in identifying the inconsistencies. For core residency events, the matrix is fairly simple:

TABLE 5: BASIC EVENT CONSISTENCY MATRIX

	**Event Code→**						
**Event Prec↓**	**ENU**	**BTH**	**IMG**	**OMG**	**DTH r**	**OBE**	**.**
**ENU**	X	X	X				X
**BTH**	X	X	X				X
**IMG**	X	X	X			**r**	X
**OMG**	X	X		X	**?**		X
**DTH**	X	X	X	X	X		X
**OBE**	X	X	X	X	X	X	X
**.**			**c**	X	X	X	X

The crossed cells indicate errors: where there is a consistent recording of events, there should be no observations in these cells. The cell with a question marks suggest that deaths might be reported by proxy respondents after out-migration, but this is highly improbable and should be checked against the original data files. All other cells will be populated with varying numbers of observations. To note, in cohort studies there should be no missing events before IMG (cell marked with "c") since observation of all individuals starts with BTH or ENU, depending on whether the cohort is a birth cohort or not. To note, in cohort studies there should be a missing event before ENU or BTH. Also, in retrospective surveys (cell and column marked with "r"), there should be no OBE after OMG, and there should be no DTH events.

To check inconsistencies, it is best to produce listing of identifiers, e.g. for cases of BTH following ENU:

```
list IndividualId Sex DoB EventDate if EventPrec==1 & EventCode==2
```

The matrix can be more complex when other events (EXT, ENT, OBS, OBL…) are added but the same interpretation applies. The cells with question marks for EXT as a preceding event indicate 'hanging cases' that should be checked against the original data files:

TABLE 6: COMPLETE EVENT CONSISTENCY MATRIX

	Event Code→										
Event Prec↓	**ENU**	**BTH**	**IMG**	**OMG**	EXT	ENT	**DTH r**	**OBE**	OBS	*OBL*	.
**ENU**	X	X	X								X
**BTH**	X	X	X								X
**IMG**	X	X	X					**r**			X
**OMG**	X	X		X		X	**?**				X
EXT	X	X	X	X	X		X	?	?	?	X
ENT	X	X	X			/					X
**DTH**	X	X	X	X	X	X	X				X
**OBE**	X	X	X	X	X	X	X	X	X	X	X
OBS	X	X									X
OBL	X	X	X	X	X	X	X	X	X	X	X
.			c	X	X	X	X	X	X	X	X

2.7 Stata programmes

Several steps are necessary to generate the core residency file described above:

- Step 1: Converting episode file into core residency file

Step 1 is not necessary if a core residency file was provided from the beginning. Check the nature of the input file first.

- Step 2: Creating a common censoring date for all individuals
- Step 3: Creating the event consistency matrix
- Step 4: Creating a residence variable

2.7.1 Step 1: Converting episode file into core residency file

```
* CONVERTING ORIGINAL "RESIDENCY EPISODE FILE" (with start events and end events)
* INTO A "CORE RESIDENCY FILE" (suitable for Event History Analysis - EHA)
* This program needs to be adapted to each site's specificities
***Create a file with only the end events
use yourresidencyfile.dta
keep IndividualId LocationId DoB Sex EndEventCode EndEventDate
* You may add variables_pertaining_to_end_events
gen file=1
rename EndEventCode EventCode
rename EndEventDate EventDate
sort IndividualId EventDate
save endeventfile, replace
***Create new file with only start events
use  residencyfile.dta, clear
keep IndividualId LocationId DoB Sex StartEventCode StartEventDate
* You may add variables_pertaining_to_start_events
gen file=2
rename StartEventCode EventCode
rename StartEventDate EventDate
sort IndividualId EventDate
save starteventfile, replace
****Append the two files (starting and ending events) to have EHA format
use starteventfile, clear
append using endevenfile
sort IndividualId EventDate EventCode
lab var EventCode "Event occurred"
label define eventlab 1 "ENU" 2 "BTH" 3 "IMG" 4 "OMG" ///
       5 "EXT" 6 "ENT" 7 "DTH" 9 "OBE" 10 "DLV", modify
lab val EventCode eventlab
* If EventDate and DoB are stored as integer variable %td or %d
* transform the format of the variable into "double": %tc
local formatdate: format EventDate
if ("`formatdate'"=="%d" | "`formatdate'"=="%td") {
       replace EventDate=cofd(EventDate)
       }

* Set all events to 12:00
replace EventDate=EventDate + (12*60*60*1000)

* Set all event that means entering the population to 06:00
* i.e. ENU, BTH, IMG
replace DoB=DoB + (6*60*60*1000)
replace EventDate=EventDate - (6*60*60*1000) ///
       if EventCode==1 | EventCode==2 | EventCode==3
* ... as well as EXT
replace EventDate=EventDate - (6*60*60*1000) ///
       if EventCode==5

* Set all event that means exiting the population to 18:00
* i.e. OMG, DTH,
replace EventDate=EventDate + (6*60*60*1000) ///
       if EventCode==4 | EventCode==7
* ... as well as ENT
```

```
replace EventDate=EventDate + (6*60*60*1000) ///
      if EventCode==6

* => baby who died on the same day they were born
* will always die 12 hours after they were born

* => people who out-migrate on the same day they in-migrated
* will always out-migrate 12 hours after they in-migrated

* Other events that do not relate to entering or exiting the population
* will be dated at 12:00, i.e. OBE, DLV, OBS, OBL, IPT, PER, AGE

order IndividualId LocationId DoB Sex EventDate EventCode
sort IndividualId EventDate
save residency, replace
```

2.7.2 Step 2: Creating a common censoring date for all individuals

```
* NB: dates stored in %tc format (millisecond)
use residency
* Here, same censoring date for all individuals (easier to manage)
* but same censoring date for each individual is sufficient
* CASE 1: Individuals still in site on 31 Dec 2010
sort IndividualId EventDate EventCode
cap drop lastrecord
bysort IndividualId: gen lastrecord=_n==_N
bysort IndividualId: gen double datebeg=cond(_n==1, DoB, EventDate[_n-1])
format datebeg %tc
lab var datebeg "Date of beginning"
*** ATTENTION: use time0(datebeg) systematically
stset EventDate, id(IndividualId) failure(lastrecord==1) time0(datebeg)
* Number of milliseconds corresponding to 1 Jan 2010
display %20.0f clock("2010.01.01 00:00:00", "YMDhms")
* 1577923200000
capture drop lastobs
stsplit lastobs, at(1577923200000)
* Drop the unnecessary observations after 31 Dec 2010 (right-censoring)
drop if EventDate>1577923200000
drop lastrecord
drop _*
* CASE 2: Individuals who died or out-migrated before 31 Dec 2010
sort IndividualId EventDate EventCode
expand=2 if IndividualId!=IndividualId[_n+1] & EventDate<1577923200000, ///
             gen(duplicate)
bysort IndividualId: replace EventDate=1577923200000 if duplicate==1
drop duplicate
* CASE 1 & 2: Last event is now OBE (=right-censoring)
bysort IndividualId: replace EventCode=9 if _n==_N
sort IndividualId EventDate EventCode
save residency.dta, replace
```

2.7.3 Step 3: Creating the consistency matrix

```
sort IndividualId EventDate EventCode
capture drop EventPrec
bysort IndividualId: gen EventPrec=EventCode[_n-1]
label define eventlab 1 "ENU" 2 "BTH" 3 "IMG" 4 "OMG" 5 "EXT" 6 "ENT" ///
      7 "DTH" 9 "OBE" 10 "DLV" 18 "OBS" 19 "OBL", modify
lab val EventCode eventlab
lab val EventPrec eventlab
set linesize 200
* To produce the Event Consistency Matrix
tab EventPrec EventCode, missing
```

2.7.4 Step 4: Creating a residence variable

```
***Create variable residence using a 6-month time criteria
* 6 months in milliseconds:
display %20.0f  (365.25 * 24 * 60 * 60 * 1000)/2
* 15778800000
```

```
capture drop residence
gen residence=.

***ENUMERATED
sort IndividualId EventDate EventCode
bysort IndividualId: replace residence=0 if EventCode==1 & (EventCode[_n+1]!=1)
***BIRTH
replace residence=0 if residence==. & EventCode==2
***EXIT
replace residence=1 if residence==. & EventCode==5
***DEATH
replace residence=1 if residence==. & EventCode==7
***ENTRY
**Cases with ENTRY preceded by EXIT and less than 180-day duration
bysort IndividualId: replace residence=1 if residence==. & ///
       (EventCode==6 & EventCode[_n-1]==5 & EventDate-EventDate[_n-1]<=15778800000)
**Cases with ENTRY preceded by EXIT and duration of more than 180 days
bysort IndividualId: replace residence=0 if residence==. & ///
       (EventCode==6 & EventCode[_n-1]==5 & EventDate-EventDate[_n-1]>15778800000)

**Cases with ENTRY preceded by OMG and less than 180-day duration
bysort IndividualId: replace residence=1 if residence==. & ///
       (EventCode==6 & EventCode[_n-1]==7 & EventDate-EventDate[_n-1]<=15778800000)
**Cases with ENTRY preceded by OMG and duration of more than 180 days
bysort IndividualId: replace residence=0 if residence==. & ///
       (EventCode==6 & EventCode[_n-1]==7 & EventDate-EventDate[_n-1]>15778800000)
**Cases of ENTRY as a first event (clearly a reconciliation issue)
bysort IndividualId: replace residence=0 if residence==. & ///
       EventCode==6 & _n==1

***IN-MIGRANT
**Cases with IMG preceded by OMG and less than 180-day duration
bysort IndividualId: replace residence=1 if residence==. & ///
       (EventCode==3 & EventCode[_n-1]==4 & EventDate-EventDate[_n-1]<=15778800000)
**Cases with IMG preceded by OMG and duration of more than 180 days
bysort IndividualId: replace residence=0 if residence==. & ///
       (EventCode==3 & EventCode[_n-1]==4 & EventDate-EventDate[_n-1]>15778800000)
bysort IndividualId: replace residence=0 if residence==. & ///
       EventCode==3 & EventCode[_n-1]==.

***OUT-MIGRANT
bysort IndividualId: replace residence=1 if residence==. & EventCode==4
by IndividualId: replace residence=0 if residence==1 & ///
       (EventCode==4 & (EventCode[_n-1]==4 | EventCode[_n-1]==3) & ///
       residence[_n-1]==0 & EventDate-EventDate[_n-1]<=15778800000)

***OBS (observation)
bysort IndividualId: replace residence=1 if residence==. & EventCode==18
* Replace by non-residence if OBS follows OMG or EXT or DTH
bysort IndividualId: replace residence=0 if residence==1 & EventCode==18 & ///
       (EventCode[_n-1]==4 | EventCode[_n-1]==5 | EventCode[_n-1]==7)

***OBL (last observation)
bysort IndividualId: replace residence=1 if residence==. & EventCode==19
* Replace by non-residence if OBL follows OMG or EXT or DTH
bysort IndividualId: replace residence=0 if residence==1 & EventCode==19 & ///
       (EventCode[_n-1]==4 | EventCode[_n-1]==5 | EventCode[_n-1]==7)

***OBE
bysort IndividualId: replace residence=1 if EventCode==9 & ///
       EventCode[_n-1]!=4 & EventCode[_n-1]!=5 & EventCode[_n-1]!=7 & ///
       EventCode[_n-1]!=19
bysort IndividualId: replace residence=0 if EventCode==9 & ///
       (EventCode[_n-1]==4 | EventCode[_n-1]==5 | EventCode[_n-1]==7 | ///
       EventCode[_n-1]==19)

* Check for cases with missing values for residence
capture drop misresidence
```

```
egen misresidence=max(residence==.), by(IndividualId)
sort IndividualId EventDate EventCode
* br IndividualId datebeg EventDate EventCode residence if misres==1

tabulate residence EventCode, missing

*** May need corrections for the remaining inconsistencies
/** Recompute indicator of missing value after each correction
cap drop misresidence
egen misresidence=max(residence==.), by(indivi)
* br IndividualId datebeg EventDate EventCode residence if misres==1

** Examples of corrections:
by IndividualId: drop if misres==1 & _n==_N & EventCode==9
bysort IndividualId: replace residence=1 if EventCode==9 & _n==_N
replace residence=1 if residence==. & misres==1 & EventCode==6
replace residence=1 if residence==. & misres==1 & EventCode==9

* May need to recode all remaining missing "residence" into 0 (=not resident)
recode residence .=0
tabulate residence EventCode, missing
*/

compress
save residency, replace
```

3 Adding events linked to ego, e.g. fertility events

The events that change the residency status of the individual form the structure of the core residency file. They are sufficient to compute birth, death, in- and out-migration rates. However, other events may be added to complete the biography of each individual: these are incidental events defined as events that do not change the individual's residency status. They may be changes in activity, marital status, socio-economic status, education, etc. One of these key biographical events is the delivery of a child.

This section uses the example of deliveries although any type of biographical event that relates to the individual could be used, e.g. field visit, hospital visit, vaccination, marriage, spouse death, spouse migration, etc. Deliveries (live births in particular) are needed to compute fertility rates and this is why we have chosen to present this as an example. To apply the procedure described below to another type of event, you just need to replace "deliveries" with the other event. If however, the event requires that the event date be imputed, it is best to refer to section 4.

Whatever the event, the crucial aspect to understand in this section is merging. Adding events that do not change residency status requires using a special form of merging, i.e. merging according to time. A command has been designed to achieve this using a Stata ado file, "tmerge.ado", with the corresponding help menu "tmerge.hlp". The Stata programmes are available in the Annexes of this document.

TABLE 7: DELIVERY CODES AND DEFINITIONS

**Individual Identifier**	**IndividualId**	**A number uniquely identifies individuals. Here, the identifier is that of the mother. The mother may not necessarily be a resident of the site.**
Individual Identifier	*FatherId*	*Optional: In case the identifier of the father is known. The father may not necessarily be a resident of the site.*
**Child identifier**	**ChildId**	**The event of a pregnancy ending after 28 weeks of gestation, which may or may not result in the birth of one or more individuals. If the delivery results in a birth of an individual, then it will be represented in the core residency file by a BTH event. If only live births have been recorded in the system, it is usually easier to extract these births from the core residency file using the condition that they can be linked to a mother and possibly a father.**
**Event**	**EventCode**	**The code is DLV (delivery) for all records**
**Event date**	**EventDate**	**The date on which the DLV (delivery) occurred**
**Child sex**	**ChildSex**	**1=Male and 2=Female**
**Multiple birth**	**MultiBirth**	**Number of children born at this delivery**
Still birth	*StillBirth*	*Optional, if recorded in the site: 0=live birth; 1=stillbirth*
Rank of delivery	RankBirth	Parity: rank of delivery for live births only
Deliver in DSA	BornDSA	Delivered in Demographic Surveillance Area (0=no; 1=yes)<br>NB: Individuals are not necessarily born to a resident mother or father
Observation date	ObservationDate	Date on which the event was observed (recorded), also known as surveillance visit date

NB: **All variables in bold font are compulsory**. Variables in normal font are advisable. *Variables in italic font are optional.*

3.1 Example of EHA file with birth history

Table 8 presents an example of an EHA file that includes deliveries of children. This file does not show the compulsory variables ChildID and ChildSex for the sake of saving space. These births are attached to identifiers of mothers, and possibly fathers. Complete birth histories might have been collected retrospectively, thus resulting in births that occurred before the onset of observation or outside the spatial limits of the site. Regardless, all births occurring within the site should be recorded. It is possible that some births in the site are attached to non-resident mothers (a child may be born to a mother who never resided in the site, admittedly a rare case). Whatever the case (births within site only or complete birth histories), all deliveries relating to mothers (and possibly fathers) who ever resided in the site should be included in the "residency event file".

TABLE 8: EXAMPLE OF AN EHA FILE THAT INCLUDES RETROSPECTIVE BIRTH HISTORIES

	IndividualId	LocationId	DoB	EventCode	EventDate	RankBirth	Counts
A	A10010010001	A100100100	17 Nov 1947	ENU	21 Aug 2002		
	A10010010001	A100100100	17 Nov 1947	OBE	31 Dec 2010		No delivery
B	A10010010002	A100100100	1 Jul 1976	ENU	21 Aug 2002		
	A10010010002	A100100100	1 Jul 1976	DLV	11 Jun 2003	**1**	1st delivery in HDSS
	A10010010002	A100100100	1 Jul 1976	DLV	31 Mar 2005	**2**	2nd delivery in HDSS
	A10010010002	A100100100	1 Jul 1976	OMG	1 Jul 2007		
	A10010010002	A100100100	1 Jul 1976	OBE	31 Dec 2010		
C	A10010010003	A100100100	23 Aug 1985	ENU	21 Aug 2002		
	A10010010003	A100100100	23 Aug 1985	EXT	1 Jul 2007		
	A10010010003	A203000104	23 Aug 1985	ENT	1 Jul 2007		
	A10010010003	A203000104	23 Aug 1985	DLV	4 Jul 2007	**1**	1st delivery in HDSS
	A10010010003	A203000104	23 Aug 1985	OMG	10 Nov 2007		
	A10010010003	A203000104	23 Aug 1985	DLV	28 Mar 2008	**2**	2nd delivery out of HDSS
	A10010010003	A100100111	23 Aug 1985	IMG	30 Mar 2009		
	A10010010003	A100100111	23 Aug 1985	DTH	15 Oct 2009	**3**	3rd delivery in HDSS
	A10010010003	A100100111	23 Aug 1985	OBE	31 Dec 2010		
D	A10010010004	A100100100	1 Jul 1988	DLV	3 Feb 1999	**1**	1st delivery out of HDSS
	A10010010004	A100100100	1 Jul 1988	DLV	19 Jul 2000	**2**	2nd delivery out of HDSS
	A10010010004	A100100100	1 Jul 1988	ENU	21 Aug 2002		
	A10010010004	A100100100	1 Jul 1988	EXT	1 Jul 2007		
	A10010010004	A203000104	1 Jul 1988	ENT	1 Jul 2007		
	A10010010004	A203000104	1 Jul 1988	DLV	3 Jul 2007	**3**	3rd delivery in HDSS
	A10010010004	A203000104	1 Jul 1988	OMG	10 May 2008		

	A10010010004	A203000104	1 Jul 1988	OBE	31 Dec 2010		
E	A10010010005	A100100100	1 Jul 2005	BTH	1 Jul 2005		
	A10010010005	A100100100	1 Jul 2005	DLV	18 Aug 2009	**1**	1st delivery in HDSS
	A10010010005	A100100100	1 Jul 2005	OBE	31 Dec 2010		
F	A10010010006	A100100100	1 Jul 1983	DLV	17 May 2005	**2**	1st delivery out of HDSS
	A10010010006	A100100100	1 Jul 1983	IMG	31 Aug 2007		
	A10010010006	A100100100	1 Jul 1983	OMG	8 Apr 2010		
	A10010010006	A100100100	1 Jul 1983	DLV	13 Jun 2010	**3**	2nd delivery out of HDSS
	A10010010006	A100100100	1 Jul 1983	OBE	31 Dec 2010		

Comments on deliveries for each of the 6 individuals A to F:

A. The simplest case: no delivery in or out of the site.
B. Most common case: all deliveries occur in the site.
C. More complex case: some deliveries occur in the site, others do not. The last delivery was simultaneous with the death of the mother. When analysing female adult mortality, it is best to artificially separate by one day (or some hours) the delivery and the maternal death (duplicate the observation and add one day or some hours to the date of death of the mother).
D. Some deliveries may have occurred before enumeration if retrospective birth histories were collected. Because delivery may occur the same day as a residential event (e.g. entry into a new household), it is best to artificially separate by one day (or some hours) the entry and the delivery.
E. Guess what is wrong here! Check for date or event inconsistencies.
F. It might be the case that no delivery actually took place within the site, delivery may even take place after out-migration, when the mother is not (or no longer) resident in the site but still on the identification system. The delivery may take place in the site while the mother was a temporary resident (e.g. she came to deliver at her own mother's home). Note that not all of a woman's deliveries may have been recorded, so the birth rank here starts at 2 for the first recorded delivery.

3.2 Procedure to add fertility events

Three steps are necessary to generate the file described above:

- Step 1: Create a duplicate of the core residency file (with no delivery events).
- Step 2: Create a file with deliveries only.
- Step 3: Merge the two files according to the individual identifier and time, including the censoring (OBE) date.

3.2.1 Step 1: Duplicate file A:

```
sort IndividualId EventDate EventCode
rename EventDate EDate
save residency2.dta, replace
```

3.2.2 Step 2: Create file B:

1. Load "residency2.dta" and keep only the variables **IndividualId** and **EDate** and the last observation with end of observation (OBE) event e.g. 31 Dec 2010. In this new file "ind_residency.dta", there should be one observation per individual as this is a file for OBE events only.

```
use residency2.dta
keep IndividualId EDate
* Number of milliseconds corresponding to 1 Jan 2010
display %20.0gc clock("2010.01.01 00:00:00", "YMDhms")
* 1,577,923,200,000
keep if EDate ==1577923200000
rename EDate dateDLV
duplicates drop IndividualId, force
sort IndividualId
save ind_residency.dta, replace
```

2. **Create a file** “deliveries.dta” containing **all deliveries** (within or outside of the HDSS), using the following specifications:
```
* Load file with IndividualId ChildId ChildDoB ChildSex ChildRank ...
use deliveries.dta, clear
rename ChildDoB dateDLV
sort IndividualId dateDLV
```
3. **Delete deliveries that occurred after end of observation (OBE) event or with missing date**.
```
drop if dateDLV>=1577923200000
drop if dateDLV==.
save deliveries.dta, replace
```
4. **Optional: In case of multiple births**, if there is not a specific record for each child, then duplicate the record and add some hours to date of birth for each subsequent births, and change the ChildId accordingly, so that each child has its own record:
```
expand =2 if MultiBirth==1, gen(duplicate)
replace dateDLV=dateDLV + 6*60*60*1000 if duplicate==1
* The following depends on the format of ChildId
replace ChilId=ChildId + ??
save deliveries.dta, replace
```
5. **Append** files “ind_residency.dta” with censoring date and “deliveries.dta”.
```
use ind_residency.dta, clear
keep IndividualId dateDLV
append using deliveries
```
6. **Delete deliveries with no parent’s identifier in file A**: these births are not supposed to have been registered in the system as deliveries (case of non-resident mothers) although they may be registered as members of the site. If there are many, check for data or programming errors. These errors may also be the result of flaws in the registration procedure.
```
egen temp=max(dateDLV==1577923200000), by(IndividualId)
drop if temp==0
drop temp
```
7. **Save sorted file under the name “deliveries.dta”.**
```
sort IndividualId dateDLV
save deliveries.dta, replace
```

3.2.3 Step 3: Merge file A and B into file C according to time using “tmerge.ado” (see Annexes):

10. **Copy the Stata programs “tmerge.ado” and “tmerge.sthlp” (or “tmerge.hlp”)** into your “…`\StataXX\ado\base\t\`” sub-directory.
11. **Merge file A and B using command “tmerge”**, replacing ‘**yoursite**’ by the name of your site:

```
tmerge IndividualId residency2(EDate) deliveries(dateDLV) yoursite_coreDLV(EventDate)
```

The new file **yoursite**`_coreDLV` will contain the core residency file with delivery events. If all individuals who have had deliveries have an end of observation OBE and if all these individuals are represented in both file A and B, there should be no error message after “tmerge”. If you do have an error message and thus cannot complete step 3, then you have to check steps 1 and 2. Do not attempt to go further if “tmerge” does not work properly.

12. Format the new EventDate as a date variable:
```
format EventDate %tc
```
13. Duplicate records that originate in both files ” (`_File==3`) and add some hours to the date of the duplicate:
```
expand =2 if _File==3, gen(duplicate)
replace EventDate=EventDate + 6*60*60*1000 if duplicate==1
sort IndividualId EventDate
```

14. Replace EventCode by 10 for "DLV" (see Table 2), when the record originates from "deliveries.dta" (`_File!=2`):
```
replace EventCode=10 if _File!=2
```
15. Replace all variables pertaining to delivery to missing when record originates from "residency2.dta" only (`_File==1`), i.e. when there was no actual delivery:
```
replace ChildSex=. if _File==1
replace ChildID="" if _File==1
replace MultiBirth=. if _File==1
replace StillBirth=. if _File==1
replace RankBirth=. if _File==1
replace BornDSA=. if _File==1
```

4 Adding other biographical events defining changes of status

Core event dates are recorded directly by the surveillance system. This is the case of birth, death, and migrations. Some other events such as delivery, field visit, hospital visit, vaccination, marriage, spouse death, spouse migration, etc., may also be recorded to the day (see previous section). However, many other events need to be reconstructed from differences in status variables recorded at different field visits. This would be the case for education status, employment status, matrimonial status, etc.

For example, education status may be recorded every three years. If a change in education level is recorded for an individual with no precise date of change, then it may be necessary to impute a date of change using the available information. Suppose a change from completed primary to completed secondary is recorded between rounds three years apart, the procedure could be to attribute a date of change using age and calendar information: the change probably occurred on the day the last school year results were announced when the individual reached 18-year old.

For other events that are not tied to a school-year or any particular seasonal calendar, it is better to attribute a mid-term date between two rounds. This would be the case of employment status or matrimonial status. The following procedure is given for this more general example.

Table 9: Change of Status Codes and Definitions

**Individual Identifier**	**IndividualId**	**A number uniquely identifies individuals.**
**Event**	**EventCode**	**The code is OBS (observation) for all records**
**Observation date**	**ObservationDate**	**Date on which the status was observed (recorded) i.e. surveillance visit date**
**Status**	**Status**	**Code for the status of interest (e.g. matrimonial status)**

NB: **All variables in bold font are compulsory**.

4.1 Example of EHA file with change of status

Table 10 presents an example of an EHA file that includes change of status at mid-term between rounds. Only changes of status between rounds are recorded, i.e. there could be several changes between rounds but only the difference between two successive rounds is recorded.

Table 10: Example of an EHA file that includes changes of status

	IndividualId	LocationId	DoB	EventCode	EventDate	Status	Comment
A	A10010010001	A100100100	17 Nov 1947	ENU	21 Aug 2002	**1**	Round 0
	A10010010001	A100100100	17 Nov 1947	OBS	11 Jul 2003	**1**	Round 1
	A10010010001	A100100100	17 Nov 1947	OBS	15 Aug 2004	**1**	Round 2
	A10010010001	A100100100	17 Nov 1947	OBS	28 Jul 2005	**1**	Round 3
	A10010010001	A100100100	17 Nov 1947	OBS	10 Sep 2006	**1**	Round 4
	A10010010001	A100100100	17 Nov 1947	OBS	04 Aug 2007	**1**	Round 5
	A10010010001	A100100100	17 Nov 1947	OBS	05 Aug 2008	**1**	Round 6
	A10010010001	A100100100	17 Nov 1947	OBS	13 Jul 2009	**1**	Round 7

	A10010010001	A100100100	17 Nov 1947	OBS	17 Sep 2010	**1**	Round 8
	A10010010001	A100100100	17 Nov 1947	OBE	31 Dec 2010	**1**	
B	A10010010002	A100100100	1 Jul 1976	ENU	21 Aug 2002	**1**	Round 0
	A10010010002	A100100100	1 Jul 1976	OBS	11 Jul 2003	**1**	Round 1
	A10010010002	A100100100	1 Jul 1976	IPT	27 Jan 2004	**1**	Mid-term between rounds
	A10010010002	A100100100	1 Jul 1976	OBS	15 Aug 2004	**2**	Round 2
	A10010010002	A100100100	1 Jul 1976	OBS	28 Jul 2005	**2**	Round 3
	A10010010002	A100100100	1 Jul 1976	OBS	10 Sep 2006	**2**	Round 4
	A10010010002	A100100100	1 Jul 1976	OMG	1 Jul 2007	**2**	
	A10010010002	A100100100	1 Jul 1976	OBE	31 Dec 2010	**2**	
C	A10010010003	A100100100	23 Aug 1985	ENU	21 Aug 2002	**1**	Round 0
	A10010010003	A100100100	23 Aug 1985	OBS	11 Jul 2003	**1**	Round 1
	A10010010003	A100100100	23 Aug 1985	OBS	15 Aug 2004	**1**	Round 2
	A10010010003	A100100100	23 Aug 1985	OBS	28 Jul 2005	**1**	Round 3
	A10010010003	A100100100	23 Aug 1985	OBS	10 Sep 2006	**1**	Round 4
	A10010010003	A100100100	23 Aug 1985	EXT	1 Jul 2007	**1**	
	A10010010003	A203000104	23 Aug 1985	ENT	1 Jul 2007	**1**	
	A10010010003	A203000104	23 Aug 1985	OBS	11 Aug 2007	**1**	Round 5
	A10010010003	A203000104	23 Aug 1985	OMG	10 Nov 2007	**1**	
	A10010010003	A100100111	23 Aug 1985	IPT	19 Jan 2008	**1**	Mid-term between rounds
	A10010010003	A100100111	23 Aug 1985	IMG	30 Mar 2009	**3**	
	A10010010003	A100100100	23 Aug 1985	OBS	13 Jul 2009	**3**	Round 7
	A10010010003	A100100111	23 Aug 1985	DTH	15 Oct 2009	**3**	
	A10010010003	A100100111	23 Aug 1985	OBE	31 Dec 2010	**3**	

Comments on changes of status for each of the 3 individuals A to C:

A. No change of status: only the dates of rounds are added to the core residency file.
B. The change of status occurs between two rounds while the individual lived in the site. In absence of exact date, the best estimation is mid-term between the two successive rounds 1 and 2, i.e. IPT = $OBS_{round1} + (OBS_{round2} - OBS_{round1})/2$. The imputed date is 27 Jan 2004 in this case.
C. The change of status occurs out of the site between out-migration and in-migration (return). No status was recorded on round 6 because the respondent was out of the site. In absence of exact date, the best estimation is mid-term between the two available rounds 5 and 7, i.e. IPT = $OBS_{round5} + (OBS_{round7} - OBS_{round5})/2$. The imputed date is 19 Jan 2008 in this case.

4.2 Procedure to add change of status

Two steps are necessary to generate the file described above:

- Step 1: Create a file with status at each round of data collection and estimate date of change of status between Rounds.
- Step 2: Merge the status file with the core residency file according to the individual identifier and time, including the censoring (OBE) date.

4.2.1 Step 1: Create file for change of status

1. Follow 3.2.1, step 1. Load "residency2.dta" and keep only the variables **IndividualId** and **EDate** and the last observation with end of observation (OBE) event e.g. 31 Dec 2010. In this new file "ind_residency.dta", there should be one observation per individual only.

```
use residency2.dta
keep IndividualId EDate
* Number of millisecond corresponding to 1 Jan 2010
display %20.0gc clock("2010.01.01 00:00:00", "YMDhms")
* 1,577,923,200,000
keep if EDate ==1577923200000
rename EDate ObservationDate
duplicates drop IndividualId, force
sort IndividualId
save ind_residency.dta, replace
```

2. **Create a file** "status.dta" containing **all recorded status** e.g. matrimonial status, education level, employment status, etc, captured during data collection rounds, using the following specifications:
```
* Load file with IndividualId ObservationDate Status
use status.dta, clear
sort IndividualId ObservationDate
```
3. **Delete records that occurred after end of observation (OBE) event or with missing date**.
```
drop if ObservationDate>=1577923200000
drop if ObservationDate==.
save status.dta, replace
```
4. **Append** files "ind_residency.dta" (created in 3.2.2 Step 2) with censoring date and "status.dta".
```
use ind_residency.dta, clear
keep IndividualId ObservationDate
append using status.dta
```
5. **Delete status records with no identifier in file A**: these changes are not supposed to have been registered for non-members of the site. If there are many, check for data or programming errors. These errors may also be the result of flaws in the registration procedure.
```
egen temp=max(ObservationDate==1577923200000), by(IndividualId)
drop if temp==0
drop temp
```
6. **Create a new record with estimated date of change of status between available rounds.** Another estimation procedure could be used depending on relevant information (e.g. school calendar for change of education status).
```
sort IndividualId ObservationDate
expand =2 if status!=status[_n+1], gen(duplicate)
sort IndividualId EventDate duplicate
replace ObservationDate=ObservationDate + ((ObservationDate[_n+1]-
ObservationDate)/2) if duplicate==1
generate EventCode=cond(duplicate==1, 20, 18)
```
7. **Save sorted file under the name "status.dta".**
```
sort IndividualId ObservationDate
save status.dta, replace
```

4.2.2 Step 2: Merge file A and B into file C according to time using "tmerge.ado" (see Annexes):

8. **If not done before, copy the Stata programs "tmerge.ado" and "tmerge.sthlp" (or "tmerge.hlp")** into your "…\StataXX\ado\base\t\" sub-directory.
9. **Merge file A and B using command "tmerge"**, replacing '**yoursite**' by the name of your site:

```
tmerge IndividualId residency2(EDate) status(ObservationDate)
yoursite_corestatus(EventDate)
```

The new file **yoursite**_corestatus will contain the core residency file with change of status. If all individuals with change of status observations have an end of observation OBE, and if all these individuals are represented in both file A and B, there should be no error message after "tmerge". If you do have an error message and thus cannot complete step 3, then you have to check steps 1 and 2. Do not attempt to go further if "tmerge" does not work properly.

10. **Format the new EventDate as a date variable:**
```
format EventDate %tc
```

5 Adding duration events

Categorising periods after or before some specific events can be extremely useful for longitudinal analysis. Often, time-to-event is period-dependent or duration-dependent. For example, the six months before delivery define a period of confirmed pregnancy. Similarly, the few months after delivery are characterised by post-partum amenorrhea, which length depends on breastfeeding and other factors. Also, the changes in economic, social, and political macro-contexts from one calendar period to the next can be superimposed on biographies to better explain individuals' behaviour. Finally, while analysis time if often age (i.e. duration since birth), duration since a particular biographical event (e.g. duration since migration, marriage or first delivery) may serve as analysis time. In that case, controlling for age (or age group) as an independent variable can be useful.

In all cases, the procedures below create time-varying covariates from the files compiled using procedures from previous sections. In other words these procedures do not necessitate extra data: they consist of handling time using data at hand only and the resulting variables are powerful tools for data analysis.

5.1 Duration specific to individual's own biography

Given the importance of migration in HDSS settings, and its potential impact on other behaviours, the following procedures are meant to create three types of variables: the first one to define periods following in-migration, the second to define time spent outside the HDSS after out-migration and before returning to the HDSS, and the third to make a distinction between new in-migrants and return migrants. The rationale would be the same to define periods before or after any other events such as deliveries, marriage or employment spells.

With regards to migration status, the respondents can only be qualified as "permanent resident" at the time of enumeration if data were collected at enumeration on the duration of residence in the HDSS area. Otherwise, one should in principle define permanent residence after some threshold, say 3 or 5 years, and begin the analysis only after 5 years running the HDSS. In practice, the uncertainty about permanent residency diminishes with time after enumeration and one can accept some uncertainty starting 3 years after enumeration. In the absence of data on duration of residence at enumeration, our advice is to impose left-censoring at least 3 years after first enumeration (round 0) as the migration variables are not reliable when the HDSS is less than 3 years-old. Therefore, in absence of precise data collected at enumeration, the "permanent resident" status is an approximation to be interpreted cautiously in the first 3 years of the HDSS and with more confidence as one moves away from the enumeration date.

TABLE 11: EXAMPLE OF A CORE RESIDENCY FILE WITH DURATION-SPECIFIC VARIABLES

	IndividualId	DoB	EventCode	EventDate	residence	IMGstatus	RETstatus	IMG_RET
A	A0010010010001	17Nov1947	ENU	21Aug2002	0	.	.	.
	A0010010010001	17Nov1947	OBE	31Dec2010	1	0	0	0
B	A0010010010002	1Jul1976	ENU	21Aug2002	0	.	.	.
	A0010010010002	1Jul1976	OMG	1Jul2007	1	0	0	0
	A0010010010002	1Jul1976	OBE	31Dec2010	0	.	.	.
C	A0010010010003	23Aug1985	ENU	21Aug2002	0	.	.	.
	A0010010010003	23Aug1985	EXT	1Jul2007	1	0	0	0
	A0010010010003	23Aug1985	ENT	2Jul2007	1	0	0	0
	A0010010010003	23Aug1985	OMG	10Nov2007	1	0	0	0
	A0010010010003	23Aug1985	IMG	30Mar2009	0	.	.	.
	A0010010010003	23Aug1985	in6m	28Sep2009	1	1	1	2
	A0010010010003	23Aug1985	DTH	15Oct2009	1	2	1	2
	A0010010010003	23Aug1985	OBE	31Dec2010	0	.	.	.
D	A0010010010004	1Jul1988	ENU	21Aug2002	0	.	.	.
	A0010010010004	1Jul1988	EXT	1Jul2007	1	0	0	0
	A0010010010004	1Jul1988	ENT	2Jul2007	1	0	0	0
	A0010010010004	1Jul1988	OMG	10May2008	1	0	0	0
	A0010010010004	1Jul1988	OBE	31Dec2010	0	.	.	.
E	A0010010010005	1Jul2005	BTH	1Jul2005	0	.	.	.
	A0010010010005	1Jul2005	OBE	31Dec2010	1	0	0	0
F	A0010010010006	1Jul1983	IMG	31Aug2007	0	.	.	.
	A0010010010006	1Jul1983	in6m	29Feb2008	1	1	2	1
	A0010010010006	1Jul1983	in2y	30Aug2009	1	2	2	1
	A0010010010006	1Jul1983	OMG	8Apr2010	1	3	2	1
	A0010010010006	1Jul1983	OBE	31Dec2010	0	.	.	.

Comments on each of the 6 individuals A to F:

A. Neither in-migration nor return migration: considered non-migrant until OBE.
B. Considered non-migrant until out-migrated from the HDSS on 1 Jul 2007. No return migration.
C. Considered non-migrant until out-migrated from the HDSS on 10 Nov 2007. Return migration on 30 Mar 2009 after less than 3 years away. Died in the HDSS 15 Oct 2009 after more than 6 months and less than 2 years following return.
D. Considered non-migrant until out-migrated from the HDSS on 10 May 2008.
E. Considered non-migrant and still in the HDSS on 31 Dec 2010.
F. In-migrant from 31 Aug 2007 after more than 3 years away since inception of HDSS in August 2002. Out-migrated after more than 2 years but less than 5 years after in-migrating.

5.1.1 Create in-migration status

In the example described above, periods 6 months, 2 years and 5 years after in-migration are defined. **Three steps** are necessary to generate the file:

- Step 1: Generate count variable of periods following in-migration.
- Step 2: Generate periods according to the duration of residence since last in-migration.
- Step 3: Re-compute all censoring variables.

5.1.1.1 Step 1: Generate count variable of periods following in-migration

```
* NB:  In-migration occurs only after period of non-residence (residence[_n-1]==0)
*      and not after internal migration (change of residence within site, i.e.
*      reconciliation of IndividualId)
cap drop count_inmig
bysort IndividualId (EventDate): ///
             gen count_inmig=sum(EventCode[_n-1]!=1 & EventCode[_n-1]!=2 ///
             & residence==1 & residence[_n-1]==0)
```

5.1.1.2 Step 2: Generate periods according to the duration of residence since last in-migration

```
* Set the analysis time to duration of residence since last in-migration only
sort IndividualId count_inmig EventDate
cap drop IndividualId_inmig
* Create a new identifier combining individual ID and period after in-migration
* NB: format the new variable as "double"
gen double IndividualId_inmig=IndividualId*100 + count_inmig
* Compute time at in-migration for each period after in-migration
cap drop time_inmig
bysort IndividualId_inmig : gen time_inmig=datebeg[1] if count_inmig>0
format time_inmig %tc
* Split duration at 6 months, 2 years & 5 years after in-migration
* NB: 6 months = minimum duration for residence
cap drop censor_death
gen censor_death=(EventCode==7) if residence==1
stset EventDate, id(IndividualId_inmig) failure(censor_death==1) time0(datebeg) ///
                   origin(time_inmig) scale(31557600000) if(residence==1)
capture drop IMG_st
stsplit IMG_st if count_inmig>0, at(0.5 2 5)
sort IndividualId EventDate
bysort IndividualId: replace EventCode=23 if EventCode==EventCode[_n+1] ///
       & IMG_st==0 & IMG_st!=IMG_st[_n+1] & IndividualId==IndividualId[_n+1]
bysort IndividualId: replace EventCode=24 if EventCode==EventCode[_n+1] ///
       & IMG_st==0.5 & IMG_st!=IMG_st[_n+1] & IndividualId==IndividualId[_n+1]
bysort IndividualId: replace EventCode=25 if EventCode==EventCode[_n+1] ///
       & IMG_st==2 & IMG_st!=IMG_st[_n+1] & IndividualId==IndividualId[_n+1]
label define eventlab 23 "in6m" 24 "in2y" 25 "in5y", modify
lab val EventCode eventlab
capture drop IMGstatus
recode IMG_st (0=1 "in-mig 0-6m") (.5=2 "in-mig 6m-2y") (2=3 "in-mig 2y-5y") ///
       (5=4 "in-mig 5y+") (.=0 "permanent res.") if residence==1, gen(IMGstatus)
label(IMGstatus)
lab var IMGstatus "In-Migrant status"
capture drop IMG_st
capture drop IndividualId_inmig
capture drop count_*
capture drop time_*
```

5.1.1.3 Step 3: IMPORTANT: Censoring variables have to be recomputed after each stsplit

```
sort IndividualId EventDate EventCode
cap drop censor_death
gen censor_death=(EventCode==7) if residence==1
cap drop censor_CoD_category
gen censor_CoD_category=cond(censor_death==1,CoD_category,censor_death)
lab val censor_CoD_category Cause_Category
* NB:  Because we stset with EventDate and time0(datebeg),
*      neither EventDate nor datebeg need to be recomputed
```

5.1.2 Create duration of residence out of the HDSS

To illustrate the procedure, we use a 3-year cut-off for the duration of residence out of the HDSS. A 3-year cut-off is only relevant if the HDSS has been run for some years (say, more than 5 years) and if left-censoring is imposed at least 3 years after first enumeration (round 0) unless data were collected at enumeration on the duration of residence in the HDSS area for each enumerated respondent.

```
* Generate count variable of periods out of the HDSS for in-migrants & out-migrants
sort IndividualId EventDate
cap drop count_out
bysort IndividualId: gen count_out=sum(residence==0 & residence[_n-1]==1)
* Generate periods according to the duration of residence since last out-migration
or since inception of the HDSS (round 0)
* Set the analysis time to duration of residence out of the HDSS
sort IndividualId count_out EventDate
* create new IndividualId for each period out of HDSS
cap drop IndividualId_out
```

```
* Don't forget to format the new variable as "double"!
gen double IndividualId_out=IndividualId*100 + count_out
cap drop time_out
bysort IndividualId_out : gen time_out=datebeg[1] if count_out>0
* Count the cumulated number of years spent outside
stset EventDate, id(IndividualId_out) failure(censor_death==1) time0(datebeg) ///
                   origin(time_out) scale(31557600000) if(residence==0)
sort IndividualId EventDate
capture drop years_out
gen years_out=_t
* Compute duration in years out of HDSS from last record before in-migration
capture drop dummy
bysort IndividualId: gen dummy=cond(residence==1 & residence[_n-1]==0,years_out[_n-
1],.)
capture drop exp_out
bysort IndividualId_inmig (EventDate): gen exp_out= ///
             sum(dummy) if residence==1 & IMGstatus>0
capture drop RETstatus
recode exp_out (0/2.99999=1 "in-mig <3y out") (3/max=2 "in-mig >3y out") ///
                    (.=0 "permanent res."), gen(RETstatus)
lab var RETstatus "Out-migration experience (3y)"
lab var exp_out "Years in out-migration"
replace exp_out=0 if exp_out==. & IMGstatus==0

capture drop dummy
capture drop IndividualId_out
capture drop count_*
capture drop time_*
```

5.1.3 Create a variable identifying new in-migrant and return migrant

This is very useful to control for previous exposure of migrants in the HDSS. Remember the limitation due to the lack of data collected on duration of residence in the HDSS area at the time of enumeration.

```
capture drop IMG_RET
gen IMG_RET=cond(count_inmig>=count_out & count_out!=0,2,cond(migr_st_exp==0,0,1))
if residence==1
lab def IMG_RET 0 "permanent res." 1 "in-migrant" 2 "return migrant", modify
lab val IMG_RET IMG_RET
```

5.1.4 Create a variable combining the different status

```
* Combine in-migration status and experience out
capture drop migr_st_exp
gen migr_st_exp=IMGstatus*10 + RETstatus
lab def migr_st_exp 0 "permanent res." ///
                   11 "6m -short exp" 12 "6m -long exp" ///
                   21 "<2y -short exp" 22 "<2y -long exp" ///
                   31 "<5y -short exp" 32 "<5y -long exp" ///
                   41 "5y+ -short exp" 42 "5y+ -long exp", modify
lab val migr_st_exp migr_st_exp

* This variable is relevant for return migrant only so combine in new variable:
capture drop migr_st_exp2
gen migr_st_exp2=cond(IMG_RET==1,IMGstatus,migr_st_exp)
lab def migr_st_exp2 0 "permanent res." 1 "<6m in-migrant" 2 "<2y in-migrant" ///
                   3 "<5y in-migrant" 4 "5y+ in-migrant" ///
                   11 "6m return-short exp" 12 "6m return-long exp" ///
                   21 "<2y return-short exp" 22 "<2y return-long exp" ///
                   31 "<5y return-short exp" 32 "<5y return-long exp" ///
                   41 "5y+ return-short exp" 42 "5y+ return-long exp", modify
lab val migr_st_exp2 migr_st_exp2
* One may choose to consider migrants to fall into the permanent resident category
after 5 years of residence:
Recode migr_st_exp2 41 42=0
```

5.2 Calendar periods

Changes in economic, social, and political macro-contexts can be defined by the transition from one calendar period to the next. One may divide calendar time into 5-year periods or decades, or one may choose other non-monotonic grouping depending on the relevant context. For some analyses, dividing calendar time into seasons or any sub-year divide can be very useful, especially if one can relate these intervals with some indicators (average temperature, rainfall, etc.).

TABLE 12: EXAMPLE OF AN EHA FILE THAT INCLUDES CALENDAR PERIODS

	IndividualId	LocationId	DoB	EventCode	EventDate	residence	period
A	A10010010001	A100100100	17 Nov 1947	ENU	21 Aug 2002	**0**	**.**
	A10010010001	A100100100	17 Nov 1947	PER	01 Jan 2004	**1**	**2000**
	A10010010001	A100100100	17 Nov 1947	PER	01 Jan 2008	**1**	**2004**
	A10010010001	A100100100	17 Nov 1947	OBE	31 Dec 2010	**1**	**2008**
B	A10010010002	A100100100	1 Jul 1976	ENU	21 Aug 2002	**0**	**.**
	A10010010002	A100100100	1 Jul 1976	PER	01 Jan 2004	**1**	**2000**
	A10010010002	A100100100	1 Jul 1976	OMG	1 Jul 2007	**1**	**2004**
	A10010010002	A100100100	1 Jul 1976	OBE	31 Dec 2010	**0**	**.**
C	A10010010003	A100100100	23 Aug 1985	ENU	21 Aug 2002	**0**	**.**
	A10010010003	A100100100	23 Aug 1985	PER	01 Jan 2004	**1**	**2000**
	A10010010003	A100100100	23 Aug 1985	EXT	1 Jul 2007	**1**	**2004**
	A10010010003	A203000104	23 Aug 1985	ENT	1 Jul 2007	**1**	**2004**
	A10010010003	A203000104	23 Aug 1985	OMG	10 Nov 2007	**1**	**2004**
	A10010010003	A100100111	23 Aug 1985	IMG	30 Mar 2009	**0**	**.**
	A10010010003	A100100111	23 Aug 1985	DTH	15 Oct 2009	**1**	**2008**
	A10010010003	A100100111	23 Aug 1985	OBE	31 Dec 2010	**0**	**.**

Comments on changes in status for each of the 3 individuals A to C:

A. The dates 1 January 2004 and 2008 are added to the core residency file. The respondent is exposed in the period 1 January 2000 - 31 December 2003, in the period 1 January 2004 – 31 December 2007, and in the period 1 January 2008 – 31 December 2011.
B. Only the date 1 January 2004 is added to the core residency file because the respondent out-migrated before 1 January 2008, i.e. in the period 2004-2007.
C. Only the date 1 January 2004 is added to the core residency file because the respondent out-migrated before 1 January 2008. The period commencing 1 January 2008 is not added because the respondent was out of the site on that date, however, the respondent is considered in the period 2008-2011 following a return on 30 March 2009 until death on 15 October 2009.

5.2.1 Split time into calendar period

The following illustrates the procedure for splitting time into calendar periods.

```
* Declare data to be suitable for EHA using calendar time as analysis time
stset EventDate if residence==1, id(IndividualId) failure(censor_death==1)
time0(datebeg)

* to get the exact value for 1 January a given year
di %20.0g tc(01Jan2000 00:00:00)
* to get the exact value for 1 January of each year
forval year=1992/2013 {
      di %20.0g tc(01Jan`year' 00:00:00)
}
* to split observation time at 1 January 2000, 2004, 2008 and 2012
capture drop period
stsplit period, at( ///
      1262304000000,  ///
      1388534400000,  ///
      1514764800000,  ///
      1640995200000)
recode period ///
      1262304000000=2000  ///
```

```
        1388534400000=2004  ///
        1514764800000=2008  ///
        1640995200000=2012
label variable period period

compress
sort IndividualId EventDate
by IndividualId: replace EventCode=20 if EventCode==EventCode[_n+1] ///
        & period!=. & period!=period[_n+1] & IndividualId==IndividualId[_n+1]
label define eventlab 30 "Period", modify
lab val EventCode eventlab
```

5.2.2 IMPORTANT: Censoring variables have to be recomputed after each stplit

```
sort IndividualId EventDate EventCode
cap drop censor_death
gen censor_death=(EventCode==7) if residence==1
cap drop censor_CoD_category
gen censor_CoD_category=cond(censor_death==1,CoD_category,censor_death)
lab val censor_CoD_category Cause_Category
* NB:  Because we stset with EventDate and time0(datebeg),
*      neither EventDate nor datebeg need to be recomputed
```

5.3 Age groups

The rationale for age groups is the same as for periods except that age (duration since birth) is used instead of calendar time. Here also the intervals may not necessarily be constant.

TABLE 13: EXAMPLE OF AN EHA FILE THAT INCLUDES LARGE AGE GROUPS

	IndividualId	LocationId	DoB	EventCode	EventDate	residence	age_group
A	A10010010001	A100100100	17 Nov 1947	ENU	21 Aug 2002	**0**	**.**
	A10010010001	A100100100	17 Nov 1947	AGE	16 Nov 2002	**1**	**50**
	A10010010001	A100100100	17 Nov 1947	AGE	17 Nov 2007	**1**	**55**
	A10010010001	A100100100	17 Nov 1947	OBE	31 Dec 2010	**1**	**60**
B	A10010010002	A100100100	1 Jul 1976	ENU	21 Aug 2002	**0**	**.**
	A10010010002	A100100100	1 Jul 1976	AGE	1 Jul 2006	**1**	**25**
	A10010010002	A100100100	1 Jul 1976	OMG	1 Jul 2007	**1**	**30**
	A10010010002	A100100100	1 Jul 1976	OBE	31 Dec 2010	**0**	**.**
C	A10010010003	A100100100	23 Aug 1985	ENU	21 Aug 2002	**0**	**.**
	A10010010003	A100100100	23 Aug 1985	AGE	23 Aug 2005	**1**	**15**
	A10010010003	A100100100	23 Aug 1985	EXT	1 Jul 2007	**1**	**20**
	A10010010003	A203000104	23 Aug 1985	ENT	1 Jul 2007	**1**	**20**
	A10010010003	A203000104	23 Aug 1985	OMG	10 Nov 2007	**1**	**20**
	A10010010003	A100100111	23 Aug 1985	IMG	30 Mar 2009	**0**	**.**
	A10010010003	A100100111	23 Aug 1985	DTH	15 Oct 2009	**1**	**20**
	A10010010003	A100100111	23 Aug 1985	OBE	31 Dec 2010	**0**	**.**

Comments on changes in status for each of the 3 individuals A to C:

A. The dates 16 November 2002 (not exact birthday due to approximation in years) and 17 November 2007 are added to the core residency file. The respondent is in the age group 50-54 until 16 November 2002, in the age group 55-59 until 17 November 2007 and in the age group 60-64 thereafter.
B. Only the date 1 July 2006 is added to the core residency file. The respondent is in the age group 25-29 until 1 July 2006 and in the age group 30-34 until out-migration.
C. Only the date 23 August 2005 is added to the core residency file. The respondent is in the age group 15-19 until 23 August 2005 and in the age group 20-24 until death on 15 October 2009.

5.3.1 Split time into age group

The following illustrates the procedure for splitting time into age groups.

```
* Declare data to be suitable for EHA using birth as origin time
* origin() defines the beginning of analysis time
* scale() transform the scale of analysis time to years
stset   EventDate   if   residence==1,   id(IndividualId)   failure(censor_death==1)
origin(time DoB) time0(datebeg) scale(365.25*24*60*60*1000)

* to split observation time at age 1, 5 and then by step of 5 up to 70
* (these ages are not exact but approximated birthdays)
capture drop age_group
stsplit age_group, at(1,5(5)70)
label def age_group 0 "<1" 1 "1-4" 5 "5-9" 10 "10-14" 15 "15-19" 20 "20-24" ///
      25 "25-29" 30 "30-34" 35 "35-39" 40 "40-44" 45 "45-49" 50 "50-54" ///
      55 "55-59" 60 "60-64" 65 "65-69" 70 "70+"
Lab var age_group age_group

compress
sort IndividualId EventDate
by IndividualId: replace EventCode=30 if EventCode==EventCode[_n+1] & ///
      age_group!=. & age_group!=age_group[_n+1] & IndividualId==IndividualId[_n+1]
label define eventlab 40 "AGE", modify
lab val EventCode eventlab
```

5.3.2 IMPORTANT: Censoring variables have to be recomputed after each stplit

```
sort IndividualId EventDate EventCode
cap drop censor_death
gen censor_death=(EventCode==7) if residence==1
cap drop censor_CoD_category
gen censor_CoD_category=cond(censor_death==1,CoD_category,censor_death)
lab val censor_CoD_category Cause_Category
* NB: Because we stset with EventDate and time0(datebeg),
*     neither EventDate nor datebeg need to be recomputed
```

6 Appendix

6.1 tmerge program

```
*! version 3.0 11/11/93 by Philippe Bocquier
* As you can see, this is not new!
* The very first version dates back from 1990
* when I was working on my PhD thesis (defended 02/11/1992)

* revised 1/04/2004 for Stata 8
* So far, it was not necessary to update to higher version. That being said,
* the syntax could probably be improved using recent Stata version.
program define tmerge
        version 3.0
        capture describe, short
        if _result(1)!=0 | _result(2)!=0 {
                error 18 /* No file should be loaded in memory */
        }
* The syntax is (below are the word identifiers attributed by the parse command):
* tmerge identifier_var file_nameA(time_varA) file_nameB(time_varB) new_file_name(new_time_var)
*              `1'  `2' `3'  `4'  `5'   `6'   `7'  `8'  `9'    `10'   `11'   `12'  `13'
        parse "`*'", parse(" ()")
* This is a basic check that the command has the right number of words
        if "`13'"!=")" | "`14'"!="" {
                di "See help tmerge"
                exit 198
        }

quietly {
        tempfile fichB fichA
        tempvar dumA dumBd dumBf error

* Use the file B
        use `6'
        capture drop _merge
* Generate a new time variable with the same value as the original time variable
* with as much precision as available (double)
        gen double `12'=`8'
* Make sure the file is well-sorted by identifier and time
        sort `1' `12'
* Generate a dummy variable being the last variable of file B
        gen byte `dumBf'=1
        save `fichB'

* Use the file A
        use `2'
        capture drop _merge
* Make it the output file
        capture save `10'
* Should be a new file or replace existing one
        if _rc!=0 {
                di " "
                noi di "File `10' already exists. Replace?" _request(oui)
                if "%oui"=="oui" | "%oui"=="o" | "%oui"=="O" | "%oui"=="Y" | "%oui"=="yes" | "%oui"=="y" {
                        save `10', replace
                }
        }
* Generate an indicator of file A
        gen byte `dumA'=1
* Generate a new time variable with the same value as the original time variable
* with as much precision as available (double)
* NB: same name as in file B
        gen double `12'=`4'
        quietly order `1'
* Make sure the file is well-sorted by identifier and time
        sort `1' `12'
* Generate a dummy variable being the last variable of file A
* (it will become the first variable of file B after merging)
        gen byte `dumBd'=1
        save `fichA'
* Merge file A with file B (keys: individual identifier & new time variable)
        capture drop _merge
        merge `1' `12' using `fichB'
        erase `fichB'
* Tricky part: reverse time
        replace `12'=-`12'
* Sort on reverse time (last record is now the first)
        sort `1' `12'

* VERY IMPORTANT: the last-become-first record (censoring date) should be the same
* in both file A and B, i.e. _merge==3
* This is because in biographical files the relevant event and date of event
* are at the end of an episode
        capture assert _merge==3 if `1'!=`1'[_n-1]
        if _rc==9 {
                noi di "Some individuals are not censored at the same time in both files."
```

```
                 noi di "Please check your files for..."
                 gen byte `error'=1-(_merge==3) if `1'!=`1'[_n-1]
                 di count if _merge==1 & `1'!=`1'[_n-1]
                 noi di "...errors (possibly no records) in `fichB' "
                 di count if _merge==2 & `1'!=`1'[_n-1]
                 noi di "...errors (possibly no records) in `fichA' "
                 noi list `1' `4' `8' _merge if `error'==1
                 drop _all
                 exit
        }
}
* NB: the simple merge above creates
* - missing values for File A variables for File B event dates
* - missing values for File B variables for File A event dates
* => need to repeat variables' values for event dates of the other file
        di "Please wait..."
* Run a sub-routine (see below) to replace the values of File A variables
* for records coming from File B (_merge==2)
        _crctmge `1'-`dumA' if _merge==2
* Run a sub-routine (see below) to replace the values of File B variables
* for records coming from File A (_merge==1)
        _crctmge `dumBd'-`dumBf' if _merge==1
        drop `dumA' `dumBd' `dumBf'
* Back to normal time
        quietly replace `12'=-`12'
        sort `1' `12'
        capture drop _File
* For more explicit names after the merge:
        rename _merge _File
        capture lab def _File 1 "`2'" 2 "`6'" 3 "both"
        lab val _File _File
        lab var _File "Record from file..."
        di " "
        di "The variable '_File' indicates in which file the time change is originated,"
        di "either `2' , `6' , or both."
        tab _File
        save `10', replace
        erase `fichA'
end

* NB: a characteristic identified by a variable is valid up to the end of an episode
* i.e. missing values have to be replaced by values of the most recent (following) record.
* (see example in tmerge.hlp)
* But it is not possible in Stata to make these replacement sequentially in reverse
* (from the last to the first record). Hence the use of reverse time where
* the value in current observation is replaced by the value of previous observation.
program define _crctmge
        version 3.0
        local varlist "req"
        local if "opt"
        parse "`*'"
        parse "`varlist'", parse(" ")
        while "`2'"!="" {
                quietly replace `2'=`2'[_n-1] `if'
                macro shift
        }
End
```

6.2 tmerge help menu

```
*! version 3.0 11/11/93 by Philippe Bocquier
To perform a match merge according to time (survival analysis)
-------------------------------------------------------------------------------

        ^tmerge^ identifier_var file_nameA^(^time_varA^)^ file_nameB^(^time_varB^)^
                        new_file_name^(^new_time_var^)^

This command merges two files both of which can contain several lines
referring to periods of time. Files A and B must contain the same
identifier_var that identifies individuals. time_varA and time_varB must
refer to the time at transition and be expressed in the same scale.
Moreover, for each individual, the time at censoring must be the same in
both files. The output file (new_file_name) will be ordered according to
new_time_var.

The command creates a new variable, _File, which indicates the file from
which the transition originated.

Example:
-------
file job                                  file mariage
id       birth   tjob     prof    id        birth    car1    tmar     car2
 2          63     80        3     2           63       1      78        4
 2          63     84        1     2           63       2      89        3
 2          63     85        2
 2          63     89        1
```

Time variables (tjob in file job, tmar in file mariage) are expressed in the same scale. In each file censoring time is 89. The program is interrupted and an error message appear if times at censoring are not the same in both files. The command could read:

```
^tmerge^ id job^(^tjob^)^ mariage^(^tmar^)^ job_mar^(^time^)^
```

The resulting file job_mar would be:

id	birth	tjob	prof	time	car1	tmar	car2	_File
2	63	80	3	78	1	78	4	mariage
2	63	80	3	80	2	89	3	job
2	63	84	1	84	2	89	3	job
2	63	85	2	85	2	89	3	job
2	63	89	1	89	2	89	3	both

NB: Original dates tjob and tmar are not modified.

The variables common to both files are stored according to the values of the first file, using the same rule as ^merge^ command.
